# Supplementary material for: Disease-associated KCNMA1 variants decrease circadian clock robustness in channelopathy mouse models
Source: J Gen Physiol. 2023 Sep 20;155(11):e202313357. doi: 10.1085/jgp.202313357 (PMC10510740; doi:10.1085/jgp.202313357)
Supplement: Table S1 — shows summary of behavioral data used in this study. [file JGP_202313357_TableS1.docx]

**Supplemental MateriaL**

Supplementary Table 1: Summary of behavioral data. Data presented as mean ± S.E.M. Period length, τ; LD, Light Dark; DD, constant darkness; FFT rPSD, fast Fourier transform relative power spectral density; α, active period; ρ, inactive period; CT16, 4-h into subjective night; +6L, 6-h phase advancement of the light:dark cycle. *Kcnma1*^WT/WT^ (n=11; 7 females, 4 males)/ *Kcnma1*^N999S/WT^ (n=14; 8 females, 6 males).  *Kcnma1*^WT/WT^ (n=11; 6 females, 5 males)/ *Kcnma1*^D434G/D434G^ (n=11; 6 females, 5 males). *Kcnma1*^WT/WT^ (n=9; 5 females, 4 males)/*Kcnma1*^H444Q/H444Q^ (n=9; 7 females, 2 males). * *P*<0.05, unpaired t-test with Welch’s correction or Mann-Whitney test.

|  | **WT** | ***Kcnma1*^N999S/WT^** |  | **WT** | ***Kcnma1*^D434G/D434G^** |  | **WT** | ***Kcnma1*^H444Q/H444Q^** |
| --- | --- | --- | --- | --- | --- | --- | --- | --- |
| **LD** | (n=11; 7F/4M) | (n=14; 8F/6M) |  | (n=11; 6F/5M) | (n=11; 6F/5M) |  | (n=9; 5F/4M) | (n=9; 2F/7M) |
| **τ (hours)** | 24.01 ± 0.01 | 23.99 ± 0.01 |  | 23.99 ± 0.01 | 23.99 ± 0.02 |  | 24.02 ± 0.01 | 24.01 ± 0.02 |
| **Χ^2^ amp** | 7043 ± 404 | 6700 ± 278 |  | 6565 ± 363 | 6371 ± 412 |  | 7177 ± 463 | 6199 ± 262 |
| **FFT** | 0.168 ± 0.01 | 0.143 ± 0.01 |  | 0.152 ± 0.02 | 0.127 ± 0.02 |  | 0.169 ± 0.02 | 0.137 ± 0.01 |
|  |  |  |  |  |  |  |  |  |
| **DD** | (n=11; 7F/4M) | (n=14; 8F/6M) |  | (n=11; 6F/5M) | (n=11; 6F/5M) |  | (n=9; 5F/4M) | (n=9; 2F/7M) |
| **τ (hours)** | 23.83 ± 0.20 | 23.71 ± 0.05 |  | 23.82 ± 0.02* | 23.60 ± 0.06* |  | 23.81 ± 0.03 | 23.80 ± 0.05 |
| **Χ^2^ amp** | 10086 ± 471* | 8271 ± 518* |  | 10440 ± 581* | 8024 ± 717* |  | 9904 ± 704 | 9071 ± 549 |
| **FFT** | 0.166 ± 0.013* | 0.107 ± 0.015* |  | 0.162 ± 0.02* | 0.107 ± 0.2* |  | 0.152 ± 0.02 | 0.131 ± 0.02 |
| **α (hours)** | 10.87 ± 0.22 | 10.27 ± 0.31 |  | 10.53 ± 0.27 | 9.82 ± 0.58 |  | 10.35 ± 0.46 | 10.37 ± 0.32 |
| **ρ (hours)** | 12.97 ± 0.23 | 13.44 ± 0.30 |  | 13.29 ± 0.26 | 13.78 ± 0.56 |  | 13.46 ± 0.45 | 13.43 ± 0.32 |
| **Bout length (mins)** | 89.69 ± 14.55 | 58.44 ± 7.54 |  | 66.12 ± 7.77 | 49.06 ± 7.97 |  | 70.51 ± 10.17 | 46.01 ± 5.79 |
| **Counts/Bout** | 4484 ± 660* | 2324 ± 328* |  | 3738 ± 605* | 1733 ± 327* |  | 3692 ± 627 | 3762 ± 1076 |
| **Bouts/Day** | 7.17 ± 0.90 | 7.57 ± 0.58 |  | 7.98 ± 0.80 | 8.51 ± 0.97 |  | 7.59 ± 0.91* | 10.56 ± 0.85* |
| **Total counts** | 27696 ± 1784* | 16650 ± 1445* |  | 26237 ± 2515* | 12541 ± 1162* |  | 23962 ± 2577 | 24940 ± 1876 |
| **α counts** | 26048 ± 1734* | 14667 ± 1432* |  | 24622 ± 2491* | 10989 ± 1106* |  | 22094 ± 2396 | 22181 ± 1877 |
| **ρ counts** | 1647 ± 124 | 1983 ± 207 |  | 1615 ± 142 | 1553 ± 175 |  | 1868 ± 338 | 2759 ± 340 |
|  |  |  |  |  |  |  |  |  |
| **Light pulse, CT16** | (n=11; 7F/4M) | (n=14; 8F/6M) |  | (n=11; 6F/5M) | (n=11; 6F/5M) |  | (n=9; 5F/4M) | (n=9; 2F/7M) |
| **Phase Shift (hours)** | -2.12 ± 0.13* | -2.75 ± 0.21* |  | -1.91 ± 0.14* | -2.67 ± 0.30* |  | -2.21 ± 0.15 | -2.28 ± 0.16 |
| **Phase Advance, +6L** | (n=11; 7F/4M) | (n=14; 8F/6M) |  | (n=11; 6F/5M) | (n=11; 6F/5M) |  | (n=9; 5F/4M) | (n=8; 2F/6M) |
| **Days to re-entrain** | 7.36 ± 0.64* | 5.29 ± 0.55* |  | 7.36 ± 0.69 | 4.00 ± 0.38 |  | 6.90 ± 0.86 | 6.50 ± 1.51 |
|  |  |  |  |  |  |  |  |  |
|  |  |  |  |  |  |  |  |  |
| **SCN neuron recordings** | (n=12; 4F/8M) | (n=14; 6F/8M) |  |  |  |  |  |  |
| **Average firing rate (Hz)** | 1.62 ± 0.37 | 2.18 ± 0.36 |  |  |  |  |  |  |
